# What Was It?

**Published:** 1874-01

**Authors:** W. L. Kirkpatrick

**Affiliations:** Cartersville, Georgia


WHAT WAS IT?—A girl just as she began to menstruate went into the creek, the flow was arrested, but ever since, some eighteen years, has had menorrhagia and frequently copious flowings, has been compelled to keep her bed largely to arrest as far as possible complete exhaustion. Two years ago I saw her and have had her under treatment for five months, she has had nearly the ordinary *flow* of health, appetite good, is able to get around smartly and is "happy." Now what is it, or was it, that is the pathological condition? I have had polypus of the uterus and everything else imaginable "on the brain, but I dont know what to call it." *It is not polypus.*

I want to say something on diarrhœa and kindred complaints sometime. I am tired and disgusted with the old and irrational therapia in the cases so everlastingly put before the profession, and all founded on a "plug" pathology, or rather no pathology at all. The "plug" don't cure. Well I am busy and can't talk now.

W. L. KIRKPATRICK, M.D.

CARTERSVILLE, GEORGIA.

["Too busy to talk!" That's the trouble. Good talkers—like our friend—leave it to a fraction of "talkers" to do all the talking, Go to talking—we want to hear you and all like you, talk.—EDS.]

